# Predictors of COVID-19 severity and outcomes in Indian patients with rheumatic diseases: a prospective cohort study

**DOI:** 10.1093/rap/rkad025

**Published:** 2023-02-28

**Authors:** Jithin Mathew, Siddharth Jain, Terence Susngi, Shankar Naidu, Varun Dhir, Aman Sharma, Sanjay Jain, Shefali Khanna Sharma

**Affiliations:** Division of Clinical Immunology and Rheumatology, Department of Internal Medicine, Postgraduate Institute of Medical Education and Research, Chandigarh, India; Division of Clinical Immunology and Rheumatology, Department of Internal Medicine, Postgraduate Institute of Medical Education and Research, Chandigarh, India; Department of Internal Medicine, Postgraduate Institute of Medical Education and Research,Chandigarh, India; Division of Clinical Immunology and Rheumatology, Department of Internal Medicine, Postgraduate Institute of Medical Education and Research, Chandigarh, India; Division of Clinical Immunology and Rheumatology, Department of Internal Medicine, Postgraduate Institute of Medical Education and Research, Chandigarh, India; Division of Clinical Immunology and Rheumatology, Department of Internal Medicine, Postgraduate Institute of Medical Education and Research, Chandigarh, India; Division of Clinical Immunology and Rheumatology, Department of Internal Medicine, Postgraduate Institute of Medical Education and Research, Chandigarh, India; Division of Clinical Immunology and Rheumatology, Department of Internal Medicine, Postgraduate Institute of Medical Education and Research, Chandigarh, India

**Keywords:** COVID-19, mortality, hospitalization, rheumatic disease, disease activity, co-morbidities

## Abstract

**Objective:**

There is dearth of data regarding the outcomes of coronavirus disease 2019 (COVID-19) among rheumatic and musculoskeletal disease (RMD) patients from Southeast Asia. We report the clinicodemographic profile and identify predictors of COVID-19 outcomes in a large cohort of Indian RMD patients.

**Methods:**

This prospective cohort study, carried out at the Postgraduate Institute of Medical Education and Research, Chandigarh (a tertiary care centre in India), included RMD patients affected with COVID-19 between April 2020 and October 2021. Demographic and clinical and laboratory details of COVID-19 and underlying RMD were noted. Predictors of mortality, hospitalization and severe COVID-19 were identified using stepwise multivariable logistic regression.

**Results:**

A total of 64 severe acute respiratory syndrome coronavirus-2-infected RMD patients [age 41.5 (19–85) years; 46 (72%) females] were included. Eighteen (28%) patients had severe COVID-19. Twenty-three (36%) required respiratory support [11 (17%) required mechanical ventilation]. Thirty-six (56%) patients required hospitalization [median duration of stay 10 (1–42) days]; 17 (27%) required intensive care unit admission. Presence of co-morbidities [odds ratio (OR) = 4.5 (95% CI: 1.4, 14.7)] was found to be an independent predictor of COVID-19 severity. Co-morbidities [OR = 10.7 (95% CI: 2.5, 45.4)] and underlying lupus [OR = 7.0 (95% CI: 1.2, 40.8)] were independently associated with COVID-19 hospitalization. Ongoing rheumatic disease activity [OR = 6.8 (95% CI: 1.3, 35.4)] and underlying diagnosis of lupus [OR = 7.1 (95% CI: 1.2, 42.4)] and SSc [OR = 9.5 (95% CI: 1.5, 61.8)] were found to be strong independent predictors of mortality. Age, sex, underlying RMD-associated interstitial lung disease and choice of immunosuppressive therapy were not associated with COVID-19 severity or adverse outcomes.

**Conclusion:**

The presence of co-morbidities was independently associated with COVID-19 severity and hospitalization. Ongoing rheumatic disease activity and the presence of lupus or SSc independently predicted mortality. Age, sex, type of immunosuppressive therapy and presence of RMD-associated interstitial lung disease did not affect COVID-19 severity or outcomes in Indian RMD patients.

Key messages

- Presence of underlying co-morbidities is an independent predictor of severe COVID-19 and related hospitalization among RMD patients.
- Ongoing rheumatic disease activity, lupus and SSc are independent predictors of COVID-19-related mortality in RMD patients.
- Age, sex, type of immunosuppressive therapy and underlying RMD-related interstitial lung disease do not influence COVID-19 severity or outcomes.

## Introduction

The coronavirus disease 2019 (COVID-19) pandemic, since its first emergence in December 2019, has become an unprecedented global health crisis, with >550 million cases reported worldwide and >6.3 million deaths [1]. It has posed unique challenges to patient care and management across all disciplines, particularly rheumatology. It was believed intuitively that patients with rheumatic and musculoskeletal diseases (RMDs) would be at a higher risk of severe COVID-19 and its complications owing to the inherent immune dysregulation, concomitant use of immunosuppressive therapy and a higher incidence of co-morbidities in this population. Despite initial conflicting data [2–5], large metanalyses have demonstrated conclusively a small but significant elevation of risk of severe acute respiratory syndrome coronavirus-2 (SARS-CoV-2) infection in this population [6, 7]. An increase in COVID-19-related mortality [odds ratio (OR) = 1.7 (95% CI: 1.1, 2.8)] in these patients was also reported in a meta-analysis by the COVID-19 Global Rheumatology Alliance (GRA); however, other measures of COVID-19 severity, such as hospitalization, oxygen supplementation and mechanical ventilation, were not significantly different in patients with or without RMDs [6]. This apparent discrepancy between the observed risk of COVID-19 infection and associated mortality without a corresponding increase in risk of surrogate outcomes might reflect a paucity of large studies assessing these outcomes rather than being a true effect estimate.

In light of these data, it is imperative to identify predictors of COVID-19 severity and outcomes among patients with RMDs to prognosticate them better and identify high-risk subgroups and factors that would need greater attention and management through a focused approach to improve COVID-19 outcomes in this vulnerable group. There is currently a lack of clarity on the determinants of adverse outcomes in these patients; data from the COVID-19 GRA and from other large-scale cohorts have reported advanced age and presence of general co-morbidities (diabetes mellitus, hypertension, chronic kidney disease and cardiovascular disease) to be most consistently associated with severe disease, hospitalization and mortality, with increased odds (ranging from 2 to 4) across most outcomes [8, 9]. However, the association of COVID-19 outcomes in these patients with other variables, such as baseline RMD diagnosis, disease activity and use of prior immunomodulators and immunosuppressants, are inconsistent across studies [10–13]. Most of these data are registry based and have a probable bias in reporting of patient data, including duplication, geographical differences and selective reporting of severe cases, which might explain the lack of consistent results.

It has been documented that ethnogeographical differences exist with respect to COVID-19 incidence and outcomes. Racial and ethnic minorities, such as African-American, Asian and Latin communities among the population in the USA with RMD, might experience a higher proportion of deaths and hospitalization with COVID-19 [14, 15]. Among the Indian population, data regarding COVID-19 incidence and outcomes in RMD patients are limited and suggest a similar incidence to the general population, with higher risk of adverse outcomes [16, 17]. However, the number of patients in these reports is limited and the severity of disease is mild, with very few patients requiring mechanical ventilation or succumbing to the illness, precluding our ability to draw conclusions on factors predictive of unfavourable COVID-19 outcomes in the Indian context.

To overcome these lacunae regarding COVID-19 in Indian RMD patients, we designed this prospective study to assess the demographic and clinical characteristics of SARS-CoV-2 infection in a cohort of RMD patients under follow-up in a tertiary care rheumatology centre in northern India. We also aimed to ascertain the predictors associated with adverse outcomes, including hospitalization, need for advanced respiratory support and mortality, in this population and to analyse these data in the context of the available literature from the western cohorts.

## Methods

### Study design and setting

This prospective cohort study was done in an in-patient and out-patient setting at the Postgraduate Institute of Medical Education and Research, Chandigarh, an apex tertiary care centre in Northern India. Consecutive patients with RMD diagnosed with COVID-19 (based on SARS-CoV-2 RT-PCR positivity) between April 2020 and October 2021 were included. The study protocol was approved by the Institutional Ethics Committee (INT/IEC/2020/SPL-1222), and the principles outlined in the 1964 Declaration of Helsinki were followed. Written informed consent was obtained from all participants before inclusion.

### Participants

Adult (age ≥18 years) patients with RMD who presented to the emergency room with RT-PCR-confirmed COVID-19 or its complications, along with those who contracted COVID-19 (identified by telephone) but who did not require hospitalization and were managed at home, were included prospectively and followed up. RMD patients under regular follow-up in the rheumatology clinic who had recovered from COVID-19 in the past were also included retrospectively. Patients <18 years of age or those unwilling to consent for participation in the study were excluded.

### Variables, their measurement and study outcomes

Demographic, clinical and laboratory data pertaining to both COVID-19 and RMD were recorded for all included patients according to a predesigned proforma. Clinical symptoms attributable to COVID-19, vital signs, treatment(s) administered for COVID-19 (antivirals and/or immunomodulatory therapies) and the final outcome (death or recovery) were noted. Severe and critical disease as defined by the World Health Organization (WHO) were grouped together as severe disease for the purpose of the present study and defined as the presence of oxygen saturation <90% on room air or signs of severe respiratory distress in addition to signs of pneumonia or presence of ARDS, sepsis, septic shock or other conditions that would normally require the provision of life-sustaining therapies, such as mechanical ventilation (invasive or non-invasive) or vasopressor therapy [18]. In addition, based on national guidelines [19], non-severe disease was categorized further as moderate disease (defined by a respiratory rate >24/min or a peripheral oxygen saturation between 90 and 94%) and as mild disease when patients with RT-PCR-confirmed SARS-CoV-2 had only upper respiratory tract symptoms without any hypoxaemia or tachypnoea. Laboratory parameters recorded included haemogram, liver function tests (total and direct bilirubin, aspartate transaminase, alanine transaminase, γ-glutamyl transferase, alkaline phosphatase and serum albumin), renal function tests, CRP, ferritin, D-dimer, fibrinogen, international normalized ratio and procalcitonin (hospitalized patients only). The neutrophil-to-lymphocyte ratio was also calculated from the differential leucocyte count. Data pertaining to the type of underlying RMD, disease activity status (active *vs* remission) at the time of acquisition of SARS-CoV-2, and ongoing treatment [supportive, NSAIDs, glucocorticoids (GCs), conventional synthetic DMARDs (including HCQ, MTX, LEF, SSZ, AZA, MMF and CYC), biologic DMARDs (rituximab or anti TNF), antifibrotics (nintedanib or pirfenidone) or IVIG] were also noted, along with details of underlying medical co-morbidities. DMARDs were also categorized into immunomodulators (MTX, LEF and SSZ) and immunosuppressants (CYC, AZA, MMF and biologic DMARDs) for the purpose of predictor analysis.

Outcomes of interest included the proportion of patients with severe COVID-19, mortality (as a percentage), hospitalization (as a percentage), intensive care unit (ICU) stay (as a percentage), the requirement for respiratory support (as a percentage) and its level (oxygen delivered by face mask or nasal prongs, non-rebreathing mask, high-flow nasal cannula, non-invasive ventilation or invasive mechanical ventilation). Predictors of COVID-19 severity, mortality and hospitalization were determined.

### Statistical analysis

Data were entered in an Excel spreadsheet and analysed using the Statistical Package for Social Sciences (v.26; SPSS, Chicago, IL, USA). The Shapiro–Wilk test was used for assessment of normality. Categorical variables were summarized as percentages or proportions and continuous variables as the mean (s.d.) or median (range) as appropriate. Categorical variables were compared using the χ^2^ or Fisher’s exact test and continuous variables using Student’s *t*-test (normally distributed data) or the Mann–Whitney *U*-test (skewed data). Age was analysed as a continuous variable and dichotomized into elderly and young based on an age cut-off of 60 years. Likewise, dose of GCs was analysed as a continuous variable and dichotomized into high dose and low dose based on a dose cut-off of 15 mg/day. Predictors of mortality, hospitalization and severe COVID-19 were identified using stepwise multivariable logistic regression analyses. Age, gender, co-morbidities, presence or absence of interstitial lung disease (ILD), type of underlying RMD, RMD disease activity status (active *vs* remission) and treatment ongoing for RMD were used as independent variables. Variables with a *P*-value < 0.2 on univariable analysis were included in the multivariable analysis. The Hosmer–Lemeshow test was used for assessment of goodness of fit of the obtained prediction models. All statistical tests were two sided, and a *P*-value < 0.05 was considered statistically significant. Consecutive sampling, with a sample size of convenience, was chosen.

## Results

### Baseline demographic and clinical data pertaining to RMD

A total of 64 patients [median age 41.5 (19–85) years; 46 (72%) females] were included. Twenty-four (38%) patients had one or more co-morbidities, including hypertension (11), diabetes (8), heart disease (4), hypothyroidism (3), chronic kidney disease (2), obstructive airway disease (2) and chronic liver disease (2); one patient each had malignancy, coeliac disease, myasthenia gravis and pancreatitis. The underlying RMD was RA in 15 (23%) patients, SLE in 12 (19%) patients, SSc in 11 (17%) patients (9 dcSSc and 2 lcSSc), idiopathic inflammatory myositis in 9 (14%) patients (anti-synthetase syndrome in six), vasculitis in six (9%) patients (large vessel vasculitis in three, one each with small, medium and variable vessel vasculitis), SpA in three patients, and UCTD and primary SS in two patients each. One patient each had APS, adult-onset Still’s disease, sarcoidosis and IgG4-related disease (Table 1). Sixteen (25%) patients had associated CTD-related ILD (CTD-ILD). Fifty-two per cent of RMD patients had active disease at the time of acquisition of COVID-19 infection. The details of ongoing RMD-related immunosuppressive and immunomodulatory treatment are provided in Table 1. Sixty-six per cent of patients were taking GCs (21% high dose, >15 mg/day) at the time of contracting SARS-CoV-2.

**Table 1.** Baseline demographic and clinical data of patients with rheumatic and musculoskeletal diseases included in the study

Parameter	Value (*n* = 64)
Age (years), mean (range)	41.5 (19–85)
Age >60 years, *n* (%)	14 (22)
Female sex, *n* (%)	46 (72)
Co-morbidities, *n* (%)	24 (38)
Underlying autoimmune rheumatic disease, *n* (%)	
RA	15 (23)
SLE	12 (19)
SSc	11 (17)
dcSSc	9 (14)
lcSSc	2 (3)
Inflammatory myositis	9 (14)
Anti-synthetase syndrome	6 (9)
Vasculitis	6 (9)
Others^a^	11 (17)
Rheumatic disease activity status, *n* (%)	
Active disease	32/61 (52)
Remission	29/61 (48)
Treatment for rheumatic disease, *n* (%)	
No/supportive treatment (including NSAIDs and anticoagulants)	6 (10)
Glucocorticoids	42 (66)
High-dose glucocorticoids (>15 mg/day)	21%
HCQ	23 (36)
Immunomodulatory therapy	
MTX	13 (20)
LEF	3 (5)
SSZ	6 (10)
Immunosuppressive therapy	
MMF	11 (17)
AZA	6 (10)
CYC	5 (8)
Rituximab	3 (5)
Etanercept	1 (2)
Others	
IVIG	2 (3)
Antifibrotic therapy	2 (3)

a SpA (*n* = 3), SS (*n* = 2), UCTD (*n* = 2), APS (*n* = 1), adult-onset Still’s disease (*n* = 1), IgG4-related disease (*n* = 1) and sarcoidosis (*n* = 1).

### Details of COVID-19-related illness and its outcomes in RMD patients

Of the 64 COVID-19 cases, 18 (28%) had severe COVID-19, 5 (8%) moderate, 37 (58%) mild and 4 (6%) asymptomatic. Twenty-three (36%) patients required respiratory support. Eleven (17%) of these required mechanical ventilation [7 (11%) invasive mechanical ventilation, 3 (5%) high-flow nasal cannula and 1 (2%) non-invasive ventilation]. Among the remaining 12 patients, three (5%) required oxygen support through a non-rebreathing mask, 8 (12%) through a venturi mask and 1 (2%) through nasal prongs. Only four (6%) patients had clinically evident thrombosis, although D-dimer was elevated in 79% of hospitalized patients. Detailed laboratory parameters of hospitalized RMD patients with COVID-19 are provided in Supplementary Table S1, available at *Rheumatology Advances in Practice* online.

Thirty-six (56%) patients required hospitalization [median duration of stay 10 (1–42) days], and 28 were managed on outpatient basis. Seventeen (27%) required ICU stay, with the median length of stay being 7 (1–30) days. The details of treatments administered for COVID-19 are provided in Table 2. Twelve (19%) patients died in the present study.

**Table 2.** Coronavirus disease-19-related outcomes in patients with rheumatic and musculoskeletal diseases

Parameter	Value (*n* = 64)
COVID-19 severity, *n* (%)	
Asymptomatic	4 (6)
Mild	37 (58)
Moderate	5 (8)
Severe	18 (28)
Respiratory support, *n* (%)	23 (36)
Mechanical ventilation	11 (17)
Invasive mechanical ventilation	7 (11)
High-flow nasal cannula	3 (5)
Non-invasive ventilation	1 (2)
Oxygen through non-rebreathing mask	3 (5)
Oxygen through venturi mask	8 (12)
Nasal prongs	1 (2)
Hospitalization, *n* (%)	36 (56)
Days of hospital stay, mean (range)	10 (1–42)
Requirement for ICU stay, *n* (%)	17 (27)
Duration of ICU stay, days, mean (range)	7 (1–30)
Death, *n* (%)	12 (19)
Treatment received for COVID-19, *n* (%)	
None	25 (39)
Glucocorticoids	20 (31)
Anticoagulation	21 (33)
Remdesivir	12 (19)
Favipravir	1 (2)
Tocilizumab	2 (3)
Immunovac/MIP	2 (3)
Azithromycin with/without vitamin C + zinc	14 (22)
Doxycycline	1 (2)
IVIG	1 (2)
Antifibrotics	1 (2)

COVID-19: coronavirus disease 2019; ICU: intensive care unit; MIP: heat-killed *Mycobacterium indicus pranii*.

### Predictors of COVID-19 severity in RMD patients

Presence of co-morbidities [OR = 4.5 (95% CI: 1.4, 14.7)] was the only factor independently associated with COVID-19 severity in our cohort of RMD patients on stepwise multivariable logistic regression (Table 3). There was no association noted with age, sex, disease activity, type of underlying RMD or its treatment, and presence of CTD-ILD.

**Table 3.** Stepwise univariable and multivariable logistic regression analysis for predictors of severe coronavirus disease 2019 in patients with rheumatic diseases

Parameter	Univariable analysis		Multivariable analysis	
	Unadjusted OR (95% CI)	*P*-value	Adjusted OR (95% CI)	*P*-value
Age, years	1.0 (1.0, 1.1)	0.36	–	–
Age > 60 years	1.0 (0.3, 3.8)	0.97		
Male sex	1.4 (0.4, 4.6)	0.56	–	–
Presence of co-morbidities	4.0 (1.3, 12.5)	0.02	**4.5 (1.4, 14.7)**	**0.01**
Active disease	1.0 (0.3, 3.1)	0.96	–	–
Diagnosis				
SLE	1.4 (0.4, 5.2)	0.66	–	–
SSc	2.6 (0.7, 9.8)	0.17	1.6 (0.3, 8.3)	0.56
RA	0.6 (0.1, 2.3)	0.43	–	–
IIM	0.3 (0.0, 2.4)	0.25	–	–
Vasculitis	1.3 (0.2, 7.9)	0.77	–	–
Presence of ILD	1.2 (0.4, 4.2)	0.75	–	–
GC	1.0 (0.3, 3.2)	1.00	–	–
High-dose GC (>15 mg/day)	1.1 (0.2, 6.9)	0.93	–	–
HCQ	0.6 (0.2, 1.9)	0.37	–	–
MTX	0.7 (0.2, 2.9)	0.62	–	–
MMF	2.5 (0.7, 9.6)	0.18	2.3 (0.4, 11.7)	0.32
Rituximab	1.3 (0.1, 14.9)	0.85	–	–
Immunomodulators	1.1 (0.3, 3.6)	0.93	–	–
Immunosuppressants	1.6 (0.5, 4.9)	0.41	–	–

The bold text indicates odds ratios and *P*-values that are significantly associated with the outcome in question.

GC: glucocorticoids; IIM: idiopathic inflammatory myopathies; ILD: interstitial lung disease; OR: odds ratio.

### Predictors of hospitalization in RMD patients infected with SARS-CoV-2

Stepwise multivariable regression analysis identified the presence of co-morbidities [OR = 10.7 (95% CI: 2.5, 45.4)] and underlying SLE [OR = 7.0 (95% CI: 1.2, 40.8)] as independent associations of hospitalization in RMD patients affected with COVID-19 (Table 4). As with severity, no association was seen with age, sex, disease activity, treatment of underlying RMD, and presence of CTD-ILD.

**Table 4.** Stepwise univariable and multivariable logistic regression analysis for predictors of hospitalization in patients with rheumatic diseases infected with coronavirus disease 2019

Parameter	Univariable analysis		Multivariable analysis	
	Unadjusted OR (95% CI)	*P*-value	Adjusted OR (95% CI)	*P*-value
Age, years	1.0 (1.0, 1.0)	0.45	–	–
Age > 60 years	0.7 (0.2, 2.4)	0.59		
Male sex	1.0 (0.3, 2.9)	0.94	–	–
Presence of co-morbidities	5.1 (1.6, 16.5)	0.006	**10.7 (2.5**, **45.4)**	**0.001**
Active disease	1.8 (0.6, 5.0)	0.27	–	–
Diagnosis				
SLE	5.0 (1.0, 25.1)	0.05	**7.0 (1.2**, **40.8)**	**0.03**
SSc	1.4 (0.4, 5.5)	0.59	–	–
RA	0.4 (0.1, 1.4)	0.15	0.6 (0.1, 4.2)	0.62
IIM	0.6 (0.1, 2.4)	0.44	–	–
Vasculitis	1.6 (0.3, 9.6)	0.59	–	–
Presence of ILD	1.0 (0.3, 3.1)	1.00	–	–
GC	1.0 (0.3, 2.9)	1.00	–	–
High-dose GC (>15 mg)	1.8 (0.3, 9.8)	0.49	–	–
HCQ	1.0 (0.3, 2.7)	0.94	–	–
MTX	0.4 (0.1, 1.3)	0.13	0.4 (0.1, 2.5)	0.32
MMF	0.9 (0.2, 3.3)	0.85	–	–
Rituximab	1.5 (0.1, 17.8)	0.73	–	–
Immunomodulators	0.6 (0.2, 1.8)	0.33	–	–
Immunosuppressants	0.7 (0.3, 2.0)	0.55	–	–

The bold text indicates odds ratios and *P*-values that are significantly associated with the outcome in question.

GC: glucocorticoids; IIM: idiopathic inflammatory myopathies; ILD: interstitial lung disease; OR: odds ratio.

### Predictors of COVID-19 mortality in RMD patients

Once hospitalized, rheumatic disease activity [OR = 6.8 (95% CI: 1.3, 35.4)] was found to be a strong independent predictor of mortality in RMD patients affected with COVID-19 (Table 5). An underlying diagnosis of SLE [OR = 7.1 (95% CI: 1.2, 42.4)] or SSc [OR = 9.5 (95% CI: 1.5, 61.8)] was independently associated with mortality, as opposed to other RMDs. Age, sex, presence of co-morbidities, underlying CTD-ILD and choice of immunomodulatory/immunosuppressive therapy were not associated with mortality in RMD patients infected with SARS-CoV-2.

**Table 5.** Stepwise univariable and multivariable logistic regression analysis for predictors of mortality in patients with rheumatic diseases infected with coronavirus disease 2019

Parameter	Univariable analysis		Multivariable analysis	
	Unadjusted OR (95% CI)	*P*-value	Adjusted OR (95% CI)	*P*-value
Age, years	1.0 (1.0, 1.0)	0.76	–	–
Age > 60 years	0.3 (0.0, 2.3)	0.23		
Male sex	0.8 (0.2, 3.5)	0.79	–	–
Presence of co-morbidities	1.2 (0.3, 4.5)	0.74	–	–
Active disease	3.4 (0.8, 14.1)	0.09	**6.8 (1.3, 35.4)**	**0.02**
Diagnosis				
SLE	2.8 (0.7, 11.3)	0.16	**7.1 (1.2, 42.4)**	**0.03**
SSc	3.2 (0.8, 13.6)	0.11	**9.5 (1.5, 61.8)**	**0.02**
RA	1.1 (0.3, 4.8)	0.89	–	–
IIM	–	1.00	–	–
Vasculitis	0.9 (0.1, 8.1)	0.89	–	–
Presence of ILD	0.5 (0.1, 2.8)	0.46	–	–
GC	0.6 (0.2, 2.3)	0.50	–	–
High-dose GC (>15 mg)	0.9 (0.1, 9.8)	0.94		
HCQ	0.8 (0.2, 3.2)	0.80	–	–
MTX	1.4 (0.3, 6.0)	0.68	–	–
MMF	1.8 (0.4, 8.1)	0.45	–	–
Rituximab	–	1.00	–	–
Immunomodulators	2.3 (0.6, 8.7)	0.21	–	–
Immunosuppressants	1.3 (0.4, 4.7)	0.68	–	–

The bold text indicates odds ratios and *P*-values that are significantly associated with the outcome in question.

GC: glucocorticoids; IIM: idiopathic inflammatory myopathies; ILD: interstitial lung disease; OR: odds ratio.

## Discussion

In this study, we present prospective data from a single cohort of RMD patients who developed COVID-19 during the first two waves in our country. Our cohort had a uniform representation of all RMDs across different age groups, and with varying severity of COVID-19. A total of 64 patients have been described, of whom 56% required hospitalization, with 19% overall mortality. RA (23%) was the commonest underlying diagnosis, followed by SLE (19%), SSc (17%), idiopathic inflammatory myositis (14%) and vasculitis (9%). To the best of our knowledge, this is the largest and most representative prospective cohort of RMD patients with COVID-19 from India. Previously published retrospective data have mostly included patients with mild, self-limited illness, with very few patients requiring advanced respiratory support or succumbing to their illness, and all the cases described were before the outbreak of the devastating second wave of illness caused by the delta variant [16, 17].

It is uncertain whether some specific autoimmune diseases predispose to SARS-CoV-2 infection and adverse outcomes more than others. CTDs are a heterogeneous group including SLE, SS, SSc, PMR, vasculitides and idiopathic inflammatory myositis etc. and have been used as a whole for comparisons with inflammatory arthritis in most studies; data on COVID-19 outcomes stratified by specific baseline RMD diagnosis are lacking. A diagnosis of RA or other forms of inflammatory arthritis has been shown to confer higher risk of SARS-CoV-2 infection, hospitalization and mortality in comparison to the general population across various studies [5, 20, 21]. Although initial trends did not highlight similar outcomes among patients with CTDs, data from subsequent larger cohorts have established the higher odds of severe COVID-19 [OR = 1.71 (95% CI: 1.06, 2.71)] and a trend towards higher mortality [OR = 1.87 (95% CI: 0.71, 4.85)] [5]. Presence of underlying SLE or SSc was an independent predictor of COVID-19-related mortality in our cohort, whereas disease severity and hospitalization were unaffected by the baseline RMD diagnosis. This might suggest possible disease-specific effects on COVID-19 mortality with respect to SLE and scleroderma in our population; however, we exercise caution in making definitive assertions about this, in view of our limited sample size. Our findings are partly in agreement with data showing an increased need for hospitalization and/or poor COVID-19 outcomes in patients with CTD in comparison to inflammatory arthritis [OR = 1.82 (95% CI: 1.0, 3.3)] [4, 22]; findings which are further supported in a large meta-analysis by Akiyama *et al.* [7].

The clinical status of underlying RMD might have an impact on COVID-19 outcomes. The proportions of patients with active disease and in remission were roughly equal in our cohort. Presence of active underlying RMD was independently associated with higher risk of COVID-19-related mortality, in agreement with prior literature [13]; the COVID-19 GRA has reported significantly poorer outcomes of COVID-19 in patients with untreated lupus and patients having moderate or high disease activity [23]. Similar findings were reported by Hasseli *et al.* among 468 RMD patients with COVID-19, with moderate to high disease activity predicting more hospitalization secondary to COVID-19 [OR = 1.96 (95% CI: 1.02, 3.76)] [13]. These findings have also been replicated in patients with primary systemic vasculitis or PMR across the large COVID-19 GRA cohort comprising ∼1200 patients [24]. Although data from GRA and other registries have little to no representation of the Southeast Asian/Indian population, limiting the generalizability of the above data, nevertheless it seems pertinent to reinforce the need to control disease activity adequately and optimize treatment in RMD patients in order to avert possible adverse COVID-19 outcomes.

Prior studies looking at demographic predictors of adverse outcomes and hospitalization in the general population have consistently reported advanced age (>65 years) and male sex as important predictors of the same [25, 26]. In patients with RMD too, advanced age is one of the most consistent risk factors for poor outcomes with COVID-19 identified across most cohorts [4, 7, 13]. The median age of patients in our study was 41.5 years, and the proportion of elderly (age > 65 years) was 16%; we did not find any association between advanced age or gender and COVID-19 severity or outcomes. We hypothesize that the lack of age predilections for adverse COVID-19 outcomes observed in our cohort might be reflective of the younger age distribution of our population in general in comparison to western cohorts, resulting in fewer numbers of elderly patients overall, precluding us from making reasonable conclusions. Although some studies have reported a male predilection for adverse COVID-19 outcomes among RMD patients [8, 13, 27], our findings are supported by a recent systematic review and meta-analysis of 83 studies, which did not find any association between sex and prevalence of COVID-19, need for mechanical ventilation, ICU admission or death [28]. The need for hospitalization was the only outcome measure positively associated with male sex across 46 studies [28]; this is in contrast to our observations and is likely to be attributable to smaller numbers.

Racial differences in COVID-19 outcomes exist, with non-White individuals, African-American, Asian and Latin communities experiencing higher proportions of deaths and hospitalization [15]. Similar findings have been replicated among racial/ethnic minorities in the USA population with rheumatic diseases [14]. The paucity of data from the Southeast Asian and the Indian populations represented in the large global GRA cohort, the lack of similar local registry data in our country and the limited observational data available with regard to COVID-19 among Indian RMD patients limit our ability to use them as comparators to put our data in perspective.

The mortality rate of 19% among RMD patients with COVID-19 in our study was higher than that observed in an Italian cohort, wherein 9% of RMD patients succumbed to COVID-19 [29]. Most population-based data report lower mortality rates, including a recent meta-analysis across 57 studies, which reported a mortality rate of only 3.5% [28]. We speculate that this higher mortality observed in the present study is attributable to a referral bias and selective inclusion of more patients with severe/critical disease requiring hospitalization and potentially missing out on mild/asymptomatic patients owing to the single-centre, tertiary care hospital-based study design.

We also observed that the presence of underlying co-morbid conditions was the sole independent predictor of COVID-19-related hospitalization [OR = 10.7 (95% CI: 2.5, 45.4)] and severity [OR = 4.5 (95% CI: 1.4,14.7)] in our cohort, but not of overall mortality. Although prior studies have reported co-morbid conditions, either individually or in combination, as key contributors to overall COVID-19 mortality among RMD patients, similar to findings in the general population [30], our findings are partly supported by the results of Wang *et al.* [28], who found that co-morbidities (hypertension, cardiovascular disease and kidney disease) were individually associated with higher risks of hospitalization, but not of overall severity or mortality. Our small sample size limits the ability to draw conclusions on the contribution of individual co-morbidities to specific COVID-19 outcomes among RMD patients.

Twenty-five per cent of patients in our study had underlying ILD, most commonly attributable to SSc, with the remainder attributable to anti-synthetase syndrome, and we observed no association with COVID-19-related hospitalization, requirement for advanced respiratory support or mortality. From the beginning of the pandemic, it was considered that people with pre-existing lung conditions, such as asthma, chronic obstructive pulmonary disease or lung cancer, were at higher risk of COVID-19 adverse outcomes [31–33]. In the subgroup of patients with ILD, Lee *et al.* [34] reported a 2.4-fold increased susceptibility to COVID-19 infection and a similar increase in susceptibility to severe outcomes with COVID-19. On further stratification of the population by type of ILD and adjustment for confounders, patients with idiopathic pulmonary fibrosis were found to be at significantly higher risk of severe COVID-19 [30], whereas CTD-ILD was associated with increased susceptibility to COVID-19 infection but not with adverse outcomes, similar to our findings. The International Severe Acute Respiratory and Emerging Infection Consortium (ISARIC) investigators have published similar data, with patients with fibrotic ILDs being most susceptible to COVID-19-related adverse outcomes once hospitalized, in comparison to CTD-related or sarcoidosis ILDs [35]. The number of CTD-ILD patients represented in both the above large cohorts are limited to 13 and 32 patients, respectively, which might limit our ability to generalize these findings to all CTD-ILDs; however, our data support the existing literature that CTD-ILD fares better than fibrotic ILDs with regard to COVID-19 outcomes.

Does baseline anti-rheumatic therapy have an effect on outcomes in RMD patients with COVID-19? Although initial data suggested varying effects of GCs, conventional synthetic DMARDs and biologic DMARDs on COVID-19 severity and outcomes [8, 11, 12], only rituximab use was consistently associated with poorer outcomes among a large retrospective cohort of hospitalized patients with COVID-19, with no evident beneficial effect of other DMARDs [36]. We report concordant results, with baseline anti-rheumatic therapy not having any significant effect on eventual COVID-19 outcomes among our RMD patients; however, only three patients in our study received rituximab, which was a limiting factor in the analysis of its effect on COVID-19 outcomes.

The limitations of our study include the following. First, the single-centre design might preclude extrapolation of our findings to the entire Indian cohort of RMD patients. Second, given that ours is a tertiary care referral centre, a bias caused by referral of sicker or more severely ill patients cannot be excluded, especially given that the mortality rates in our study were much higher than what has been reported previously [28, 29]. Third, we did not have data regarding vaccination status, reinfections and vaccination failures for these patients. All the patients in this cohort had been infected with COVID-19 during the first two waves, which meant that most of them had not been vaccinated before or would have had received only a single dose of the available vaccine by the time they were COVID positive. Fourth, the number of patients in our cohort is limited, and we might have missed out on the data of RMD patients who were under our follow-up but were admitted elsewhere for COVID-19. Lastly, we did not have data on radiological outcomes in our cohort.

In conclusion, we describe predictors of COVID-19 severity and adverse outcomes from one of the largest cohorts of RMD patients with COVID-19 from India. Inclusion of a patient population representing the entire spectrum of RMDs, with adequate representation of patients with varying severity of COVID-19 and mortality, is one of the key strengths of our study. Ongoing rheumatic disease activity, SLE and SSc were independently associated with COVID-19 mortality, whereas presence of underlying co-morbidities was associated with increasing hospitalization and severity of COVID-19. There was no association between age, gender, baseline immunomodulatory therapy, presence of ILD and eventual COVID-19 severity and outcomes. Patients with RMD constitute a vulnerable population owing to the nature of their disease and the effect of immunosuppressive medications they are taking. Identification of predictors of infectious disease-related severity and mortality in these patients can help in better risk stratification of this diverse group of disorders and in better triaging and rationing of limited health-care resources in a pandemic/emergency setting. The knowledge that patients with ongoing rheumatic disease activity and certain specific disease subgroups are predisposed to adverse outcomes, including hospitalization and death, warrants a shift of focus towards more intensive control of disease activity in these patients and their prioritization for preventive measures, such as vaccination (by virtue of inclusion in high-risk groups). Such focused approaches targeting vulnerable subgroups might help to mitigate the deleterious effects of similar infectious disease outbreaks in the future.

## Supplementary Material

rkad025_Supplementary_Data

## Data Availability

The data underlying this article are available in the article and in its online supplementary material.
