# Supplementary material for: Predictors of COVID-19 severity and outcomes in Indian patients with rheumatic diseases: a prospective cohort study
Source: Rheumatol Adv Pract. 2023 Feb 28;7(1):rkad025. doi: 10.1093/rap/rkad025 (PMC9995091; doi:10.1093/rap/rkad025)
Supplement: rkad025_Supplementary_Data [file rkad025_supplementary_data.docx]

**Supplementary Table S1: Vital signs and laboratory parameters for patients hospitalized with COVID-19**

| **Parameter** | **Value (n=36)** |
| --- | --- |
| Pulse rate (beats per min) | 99 ± 16 |
| Systolic BP   - >160 mm Hg - 140-159 mm Hg - 120-139 mm Hg - 90-119 mm Hg - <90 mm Hg | 2  3  13  16  1 |
| Respiratory rate | 24 ± 6 |
| SpO2 | 89 (74-98) |
| Hemoglobin (g/dL) | 10.2 ± 2.5 |
| Anemia^a^ | 26/34 (76%) |
| Total leucocyte count (/mm^3^) | 9433 ± 4743 |
| Leucopenia^b^ | 3/35 (8%) |
| Leucocytosis^c^ | 12/35 (34%) |
| NLR | 6.6 (0.3 -89.1) |
| Platelet count (X1000/mm^3^) | 198 (15-499) |
| Thrombocytopenia^d^ | 7/34 (21%) |
| Urea (mg/dL) | 42.5 (15.9-187) |
| Creatinine (mg/dL) | 0.8 (0.3-7.2) |
| Raised creatinine^e^ | 10/33 (30%) |
| Bilirubin (mg/dL) | 0.5 (0.1-2.8) |
| Total protein (g/dL) | 6.1 ± 0.9 |
| Albumin (g/dL) | 3.0 ± 0.8 |
| AST (U/L) | 30 (9-1155) |
| Elevated AST^f^ | 13/31 (42%) |
| ALT (U/L) | 24 (10-768) |
| Elevated ALT^g^ | 8/31 (26%) |
| ALP | 109 (44-634) |
| CRP (mg/L) | 47.8 (0.8-347.8) |
| Elevated CRP^h^ | 27/31 (87%) |
| Ferritin (ng/mL) (n=24) | 894 (59-13580) |
| Elevated ferritin^i^ | 16/24 (67%) |
| INR | 1.09 (0.89-2.21) |
| D-dimer (ng/mL) (n=24) | 754 (146-20000) |
| Elevated D-dimer^j^ | 19/24 (79%) |
| Fibrinogen (n=18) | 4.8 ± 1.8 |
| Procalcitonin (n=21) | 0.3 (0.03-36.2) |

NLR: Neutrophil lymphocyte ratio; AST: Aspartate transaminase; ALT: Alanine transaminase; CRP: C reactive protein. Data are presented as n (%), n (± SD), or n (range) where appropriate.

^a^Anemia: Hemoglobin < 12 g/dl

^b^Leucopenia: TLC < 4000/uL

^c^Leucocytosis: TLC > 11000/uL

^d^Thrombocytopenia: Platelet count <100,000/uL

^e^Raised creatinine: Creatinine> 1.3

^f^Elevated AST: AST > 40 U/L

^g^Elevated ALT: ALT > 40 U/L

^h^Elevated CRP: > 6 mg/L

^i^Elevated ferritin: >400 ng/mL

^j^Elevated D-Dimer: >240 ng/mL
